# Using a New Landmark of the Most External Point in the Embolization of Distal Anterior Choroidal Aneurysms: A Report of Two Cases

**DOI:** 10.3389/fneur.2020.00693

**Published:** 2020-07-31

**Authors:** Nai Zhang, Wen-qiang Xin, Shu-yuan Yue, Yue Zhong, Wei Wei, Xin-yu Yang, Zi-jun Wang, Fu-shun Xiao

**Affiliations:** ^1^Department of Neurosurgery, Tianjin Medical University General Hospital, Tianjin, China; ^2^Department of Neurology, Mianyang Central Hospital, Sichuan, China; ^3^Department of Science, Biology, University of Alberta, Edmonton, AB, Canada

**Keywords:** most external point, distal anterior choroidal aneurysms, plexal point, embolization, case report

## Abstract

There are landmarks on the course of the anterior choroidal artery (AChoA), such as the original point (OP) and the plexal point (PP), as documented in previous articles. In these previous articles, the AChoA was the terminal branch of the internal carotid artery (ICA), which had two segments throughout its course. The first cisternal segment began from the origin and ended at the point where the artery reached the choroidal fissure (the PP). The second segment consisted of one or more branches, which passed through the choroidal fissure and entered the choroid plexus. However, we found another angiographic landmark, named the most external point (MEP), along the course of the AChoA in the anteroposterior (AP) view. There was a sharp turn at the outermost limit of the course of the AChoA, and then the AChoA progressed inward and upward. We defined the outermost limit as the MEP of the AChoA. This study describes two rare cases of distal AChoA aneurysms associated with arteriovenous malformation (AVM) and Moyamoya disease that developed intraventricular hemorrhage, and we used the parent artery occlusion (PAO) technique to embolize the distal AChoA lesions at the MEP. The patients recovered well without any neurological complications.

## Introduction

Reports on distal anterior choroidal artery (AChoA) aneurysms are extremely rare; until now, fewer than 50 cases have been reported in the literature (1). The embolization of a distal AChoA aneurysm by the parent artery occlusion (PAO) technique is an effective therapy. Normally, neurosurgeons deem that sacrificing the distal AChoA beyond the plexal point (PP) will not cause any neurological deficit. However, it is potentially hazardous, as there are four to six perforating branches arising from the distal AChoA (2). We aimed to establish a new embolization landmark [the most external point (MEP)] to safely embolize distal AChoA lesions and to analyze the necessity of using this new embolization landmark.

## Case Report

### Case 1

**Figure 1:**
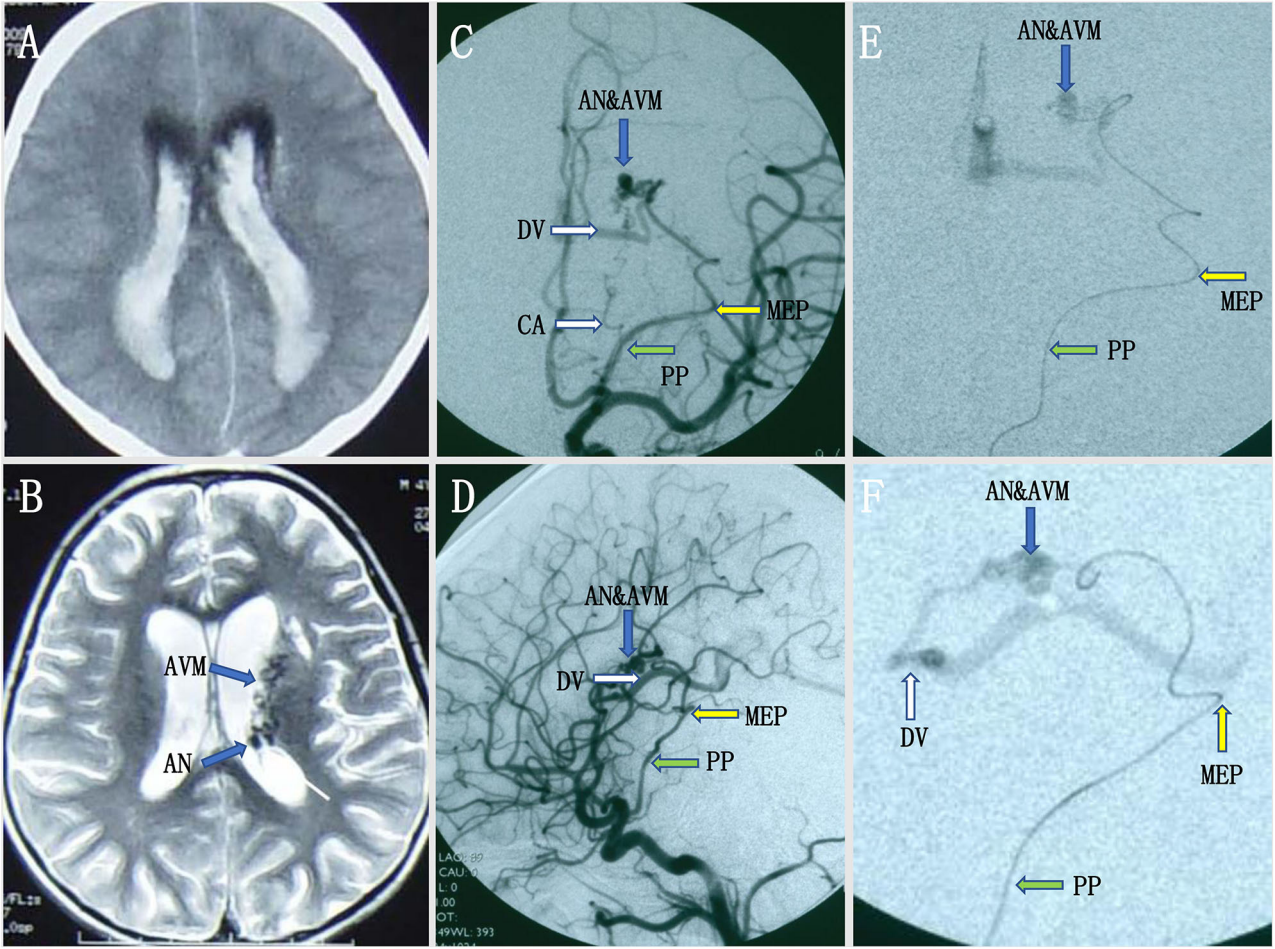
**(A,B)** Intraventricular hemorrhage was noted on initial computed tomography (CT), and subsequent magnetic resonance imaging (MRI) revealed multiple signal-void vascular structures in the lateral ventricle with enlarged aneurysmal structures. **(C,D)** Preoperative digital subtracted angiography (DSA) revealed a distal AChoA aneurysm associated with an intraventricular arteriovenous malformation (AVM) with deep venous drainage via an enlarged internal cerebral vein and the great cerebral vein of Galen into the straight sinus, visualized in the early DSA. **(E,F)** The ultra-selective DSA [anteroposterior (AP) view **(E)** and lateral view **(F)**] in the operation showed that the microcatheter had crossed the most external point (MEP) of the anterior choroidal artery (AChoA), and the microcatheter tip rested on the right point, as our planned onyx reflux point was distal to the MEP.

**Figure 2:**
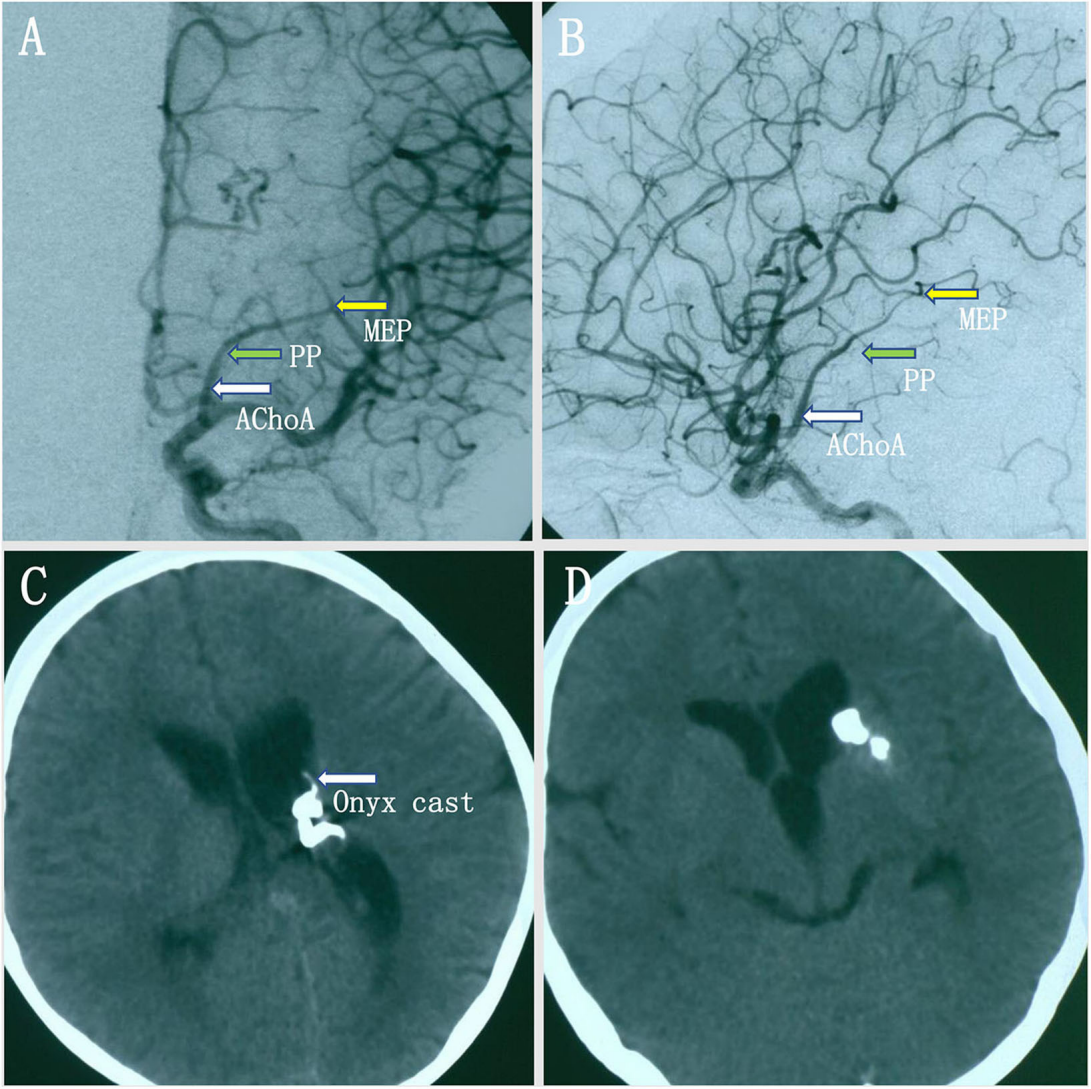
**(A,B)** Intraoperative digital subtracted angiography (DSA) showed that the anterior choroidal artery (AChoA) was embolized distal to the embolization landmark (the MEP) by Onyx, and the ipsilateral capsulothalamic artery was retained [anteroposterior (AP) **(A)** and lateral **(B)** views]. **(C,D)** Postoperative computed tomography (CT) showed that the intraventricular hemorrhage had disappeared, the distal AChoA aneurysm and associated arteriovenous malformation (AVM) was embolized by glue (Onyx-18), and the Onyx cast was at the location of the distal AChoA aneurysm and the AVM in the left ventricle.

A pediatric patient with an acute headache, vomiting, and loss of consciousness was admitted to our hospital. Intraventricular hemorrhage was noted on CT, and subsequent MRI revealed multiple signal-void vascular structures in the lateral ventricle with enlarged aneurysmal structures (Figures 1A,B). The patient began improving 25 days later and achieved good neurological recovery. A digital subtracted angiography (DSA) was conducted, and a left internal carotid artery (ICA) angiogram showed an arteriovenous malformation (AVM) with a nidus measuring 2.4 × 1.8 × 1.5 cm supplied by the collateral enlarged AChoA and the lenticulostriate arteries. Drainage was placed into the straight sinus. In addition, there was a distal AChoA aneurysm near the nidus of the AVM distal to the MEP (Figures 1C,D). We chose endovascular treatment to embolize the lesion under general anesthesia because the patient was a child. First, we deployed a microcatheter (Marathon) proximal to the aneurysm by ultra-selective DSA, and ultra-selective angiography confirmed that the microcatheter tip had navigated across the MEP, which was the correct location to occlude the AChoA by the PAO technique (Figures 1E,F). Then, the microcatheter was flushed with 0.25 ml of dimethyl sulfoxide (DMSO), and Onyx-18 was injected. Finally, the distal AChoA aneurysm and AVM disappeared, and the distal AChoA was embolized beyond the MEP by Onyx. In addition, the proximal AChoA prior to the MEP with the ipsilateral capsulothalamic artery was retained (Figures 2A,B). Post-embolization CT revealed the glue cast within the previous lesion of the preoperative head MRI (Figures 2C,D).

### Case 2

**Figure 3:**
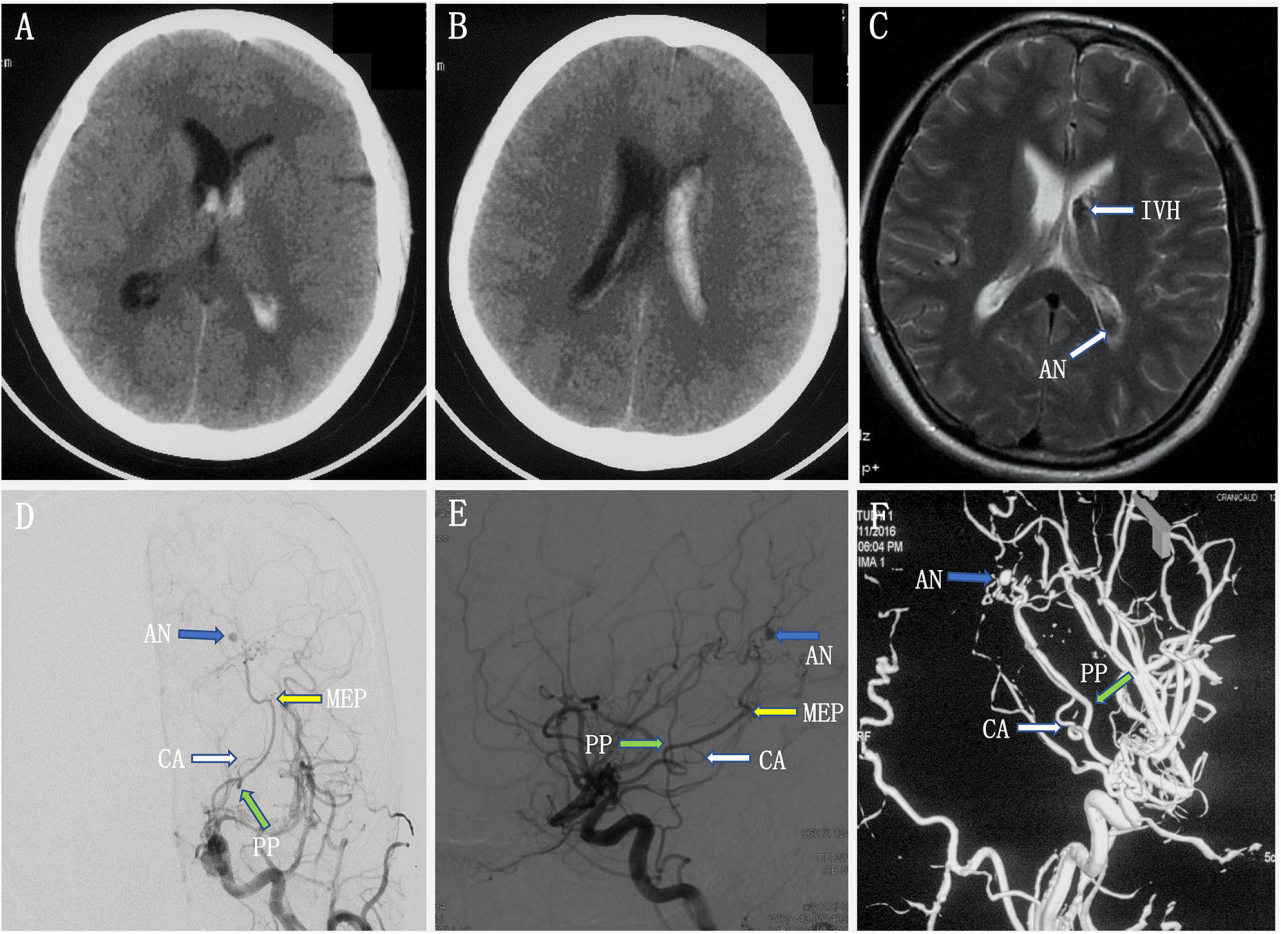
**(A–C)** Preoperative head computed tomography (CT) showed a left intraventricular hemorrhage, and axial magnetic resonance imaging (MRI) demonstrated a dilated artery and a suspected aneurysm in the left ventricle. **(D,F)** Preoperative digital subtracted angiography (DSA) [anteroposterior (AP) **(D)** and lateral **(E)** views] and 3D rotational angiographies **(F)** showed a narrowed internal carotid artery (ICA) bifurcation, A1, and M1 with Moyamoya change (Suzuki stage II–III). In addition, there was a dilated anterior choroidal artery with a distal aneurysm beyond the most external point (MEP).

**Figure 4:**
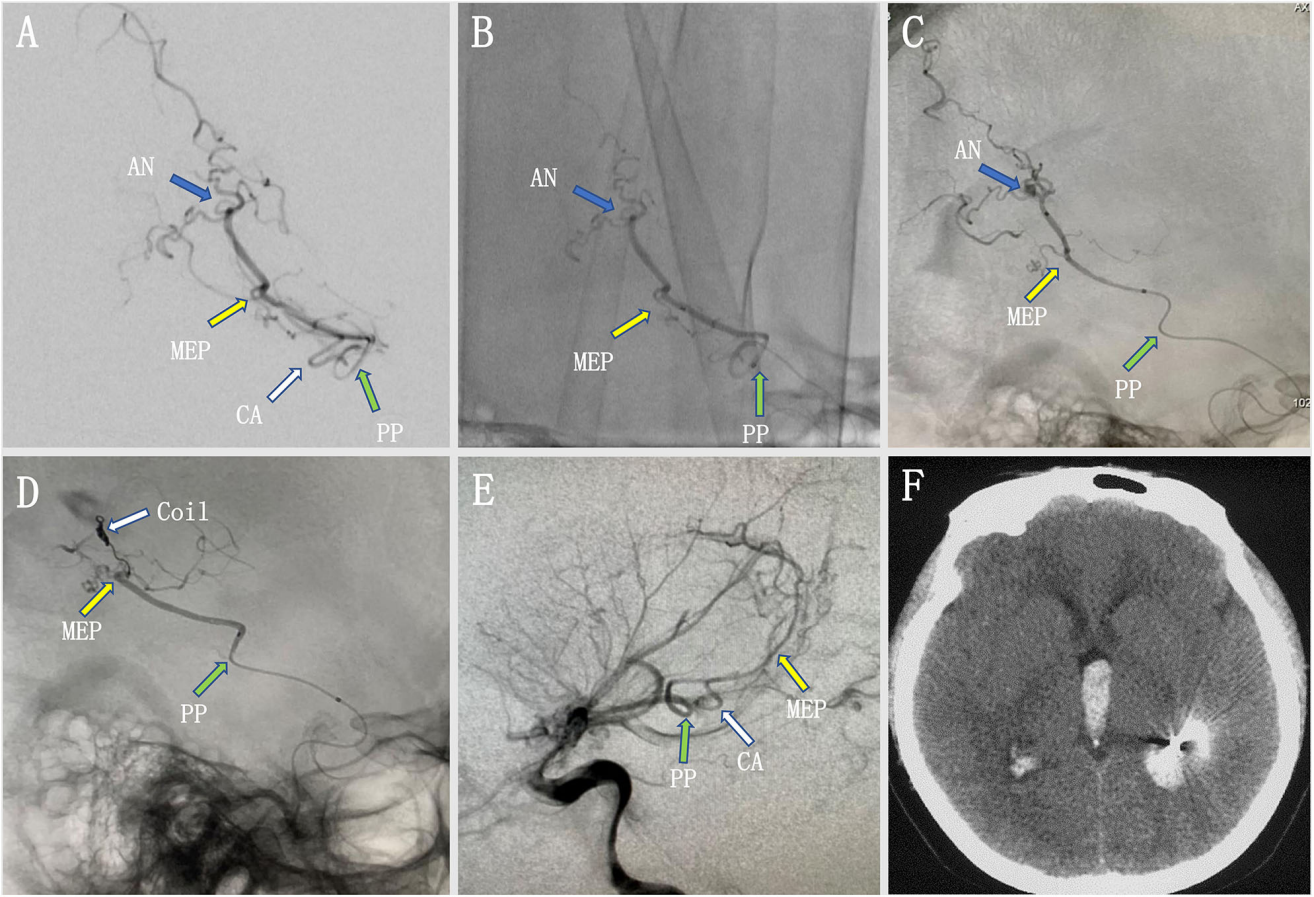
**(A–C)** Ultra-selective digital subtracted angiography (DSA) at the plexal point (PP) **(A)** in the operation showed a large capsulothalamic artery arising at the PP. The ultra-selective DSA at the most external point (MEP) **(B)** showed two other branches arising at the MEP of the anterior choroidal artery (AChoA). The ultra-selective DSA **(C)** showed the microcatheter tip had crossed the MEP and another branch arising near the distal AChoA aneurysm. Therefore, we decided to embolize the aneurysm and the distal AChoA by coils. **(D,E)** Ultra-selective DSA **(D)** showed that the distal AChoA aneurysm and the AChoA beyond the MEP were embolized by coils, and DSA of the left internal carotid artery (ICA) **(E)** showed that there was no retrograde filling of the aneurysm by collaterals and that the ipsilateral capsulothalamic artery had been retained. **(F)** Postoperative head computed tomography (CT) showed that the AChoA aneurysm was embolized beyond the MEP with coils, and the intraventricular hemorrhage had disappeared.

A young woman experienced a sudden onset of vomiting and severe headache without any neurological deficits. A head CT scan revealed intraventricular hemorrhage in the left lateral ventricle, but the lateral, third, and fourth ventricles were not enlarged (Figures 3A,B). Axial MRI showed a dilated vessel, and a suspected aneurysm was found in the left lateral ventricle (Figure 3C). Preoperative DSA showed a narrowed ICA bifurcation, A1, and M1 with Moyamoya change (Suzuki stage II–III). In addition, there was a dilated AChoA branch with a distal aneurysm beyond the MEP (Figures 3D–F). The embolization operation was performed under general anesthesia 2 weeks later, when the patient's symptoms had disappeared. First, an Echelon-10 microcatheter was deployed into the AChoA. Then, ultra-selective DSA was performed: ultra-selective DSA at the PP (Figure 4A) demonstrated a large capsulothalamic artery arising at the PP of the AChoA; ultra-selective DSA at the MEP (Figure 4B) showed two other branches arising at the MEP of the AChoA; and ultra-selective DSA (Figure 4C) beyond the MEP showed another branch arising near the aneurysm of the AChoA. Therefore, we decided to embolize the aneurysm and the distal AChoA beyond the MEP by coils and to preserve the proximal AChoA with the large capsulothalamic artery and other branch vessels to the greatest extent possible. Finally, the distal AChoA aneurysm and the AChoA beyond the MEP were embolized by coils (Axium; 2 mm × 3 cm, 2 mm × 1 cm), and DSA of the ICA showed that there was no retrograde filling of the aneurysm by collaterals and that the ipsilateral capsulothalamic artery had been retained (Figures 4D,E). Postoperative head CT revealed that the intraventricular hemorrhage had disappeared, and the coils were cast just in the location of the distal AChoA aneurysm in the occipital horn of the lateral ventricle (Figure 4F). The patient recovered well postoperatively, and 1-year follow-up examination revealed that no rebleeding had occurred.

## Discussion

As originally proposed by Rhoton et al. (3) the AChoA was the terminal branch of the ICA, which had two segments throughout its course. The first cisternal segment began from the origin and ended at the point where the artery reached the choroidal fissure (the PP). The second segment consisted of one or more branches, which passed through the choroidal fissure and entered the choroid plexus. The angiographic PP was considered the key anatomic landmark of the operation, and embolization beyond the PP was generally accepted as safe (4, 5). However, the AChoA might send off a few small recurrent perforating branches that exit the temporal lobe through the choroidal fissure to supply the optic tract, the cerebral peduncle, and the thalamus (3). Erdem et al. (6) found that the plexal segment of the AChoA passed through the choroid fissure as a single trunk and then divided into the lateral plexal and medial perforating branches within the choroid plexus in 16% of the specimens studied.

**Figure 5:**
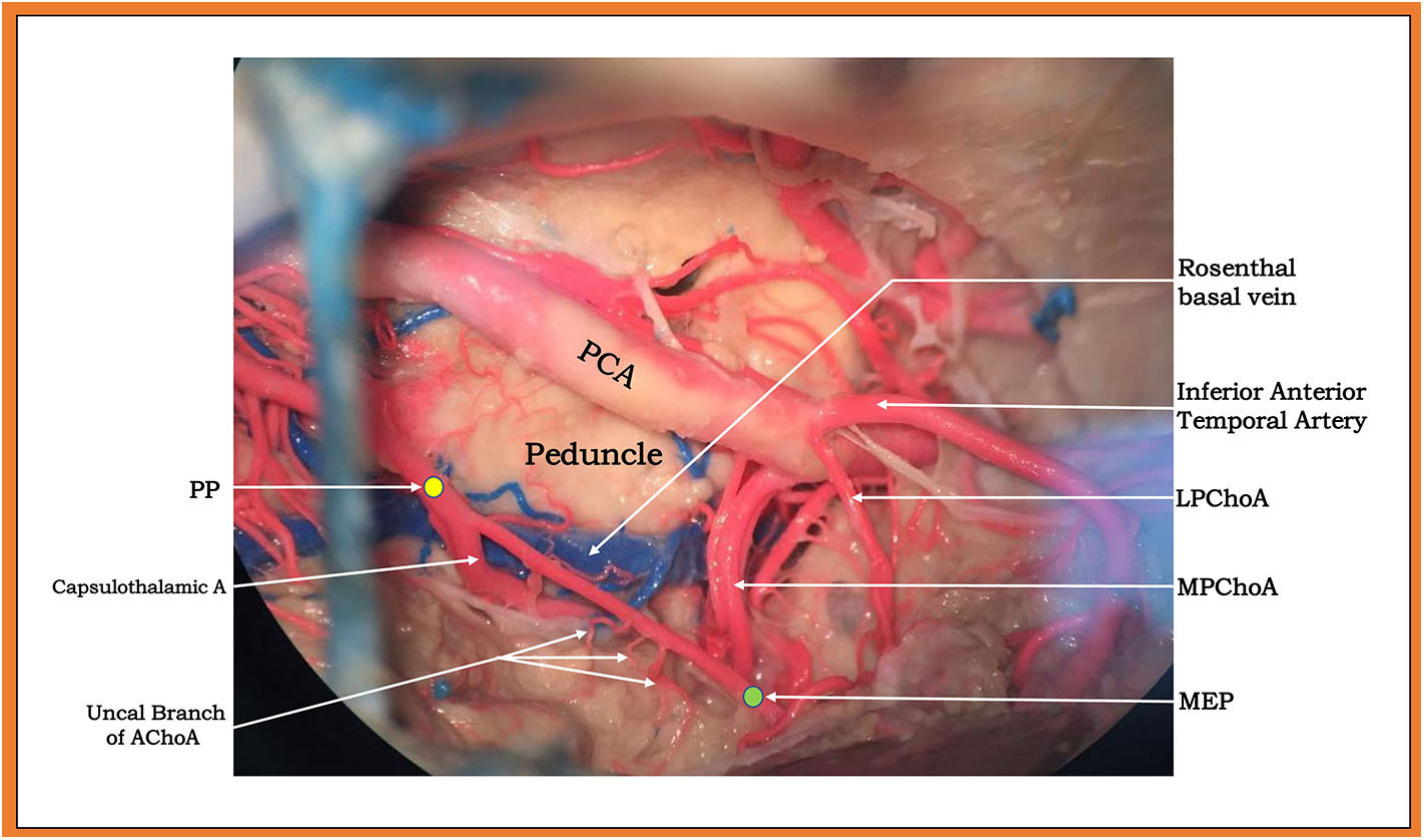
Upper and lateral view (subtemporal approach) of a large capsulothalamic artery that arises from the right anterior choroidal artery (AChoA), just rostral to the inferior horn of the lateral ventricle. The other perforators are indicated by arrows: the uncal branch of the AChoA and the peduncular branch of the AChoA.

Furthermore, we found that the plexal segment of the AChoA passed through the choroid fissure as a single trunk and sent off a large capsulothalamic perforator and a few small recurrent perforating branches that exited the temporal lobe through the choroidal fissure to supply the optic tract, cerebral peduncle, and thalamus (Figure 5). To safely embolize distal AChoA lesions, a new landmark of the AChoA, named the MEP, was identified. It could be identified easily in the frontal projection and the lateral projection by DSA, and sketches were drawn to illustrate the identification of the MEP. The MEP corresponds to the internal limit of the course of the AChoA, which then turns direction and progresses inward. The PP is usually at the midpoint of the original point (OP) and the MEP.

We analyzed the reasons why the MEP was much safer than the PP for the embolization of distal AChoA aneurysms or AVMs. First, in our two cases, we noted the most important perforating branch on the cisternal segment of the AChoA, known as the capsulothalamic artery. Consistent with the literature, the capsulothalamic artery was the most distal perforator of the AChoA, usually single, most often originating close to the PP (7). Then, we found a few small perforating branches on the plexal segment of the AChoA. As documented in publications, these small perforating branches, which the plexal segment of the AChoA gives off, may recurrently exit the temporal lobe through the choroidal fissure to supply the optic tract, the cerebral peduncle, and the thalamus (3) or important brain structures in the lateral ventricle, such as the optic radiation of Mayer's loop, which passes over the lateral aspect of the temporal horn.

## Conclusion

The MEP was an effective landmark for endovascular surgeons when treating distal AChoA lesions. However, more studies are needed to verify and integrate this theory.

## Data Availability Statement

The original contributions presented in the study are included in the article/supplementary material, further inquiries can be directed to the corresponding author/s.

## Ethics Statement

The studies involving human participants were reviewed and approved by Tianjin Medical University General Hospital ethics committee. Written informed consent to participate in this study was provided by the participants' legal guardian/next of kin. Written informed consent was obtained from the individual(s), and minor(s)' legal guardian/next of kin, for the publication of any potentially identifiable images or data included in this article.

## Author Contributions

WX and NZ designed the study, acquired the data, drafted the article, and analyzed. SY, YZ, WW, XY, ZW, and FX acquired the data and interpreted the data. All authors revised the article critically for important intellectual content together and approved the version to be published.

## Conflict of Interest

The authors declare that the research was conducted in the absence of any commercial or financial relationships that could be construed as a potential conflict of interest.

## Abbreviations

PP: plexal point
MRI: magnetic resonance imaging
ICA: internal carotid artery
MEP: most external point
PAO: parent artery occlusion
DSA: digital subtracted angiography
CT: computed tomography
AVM: arteriovenous malformation
ICA: internal carotid artery
AChoA: anterior choroidal artery.
